# An Atypical Presentation of Relapsing Polychondritis Manifesting As Acute Angle Closure

**DOI:** 10.7759/cureus.42853

**Published:** 2023-08-02

**Authors:** Hays Cape, William C Gambla, Ilyse S Kornblau

**Affiliations:** 1 Ophthalmology, The University of Tennessee Health Science Center, Memphis, USA; 2 Surgery, Veterans Affairs Medical Center, Memphis, USA

**Keywords:** vexas, choroidal effusion, glaucoma, acute angle closure, relapsing polychondritis, episcleritis, scleritis, uveitis, ocular inflammation, autoimmune disease

## Abstract

Relapsing polychondritis (RPC) is a rare autoimmune disease characterized by recurrent inflammation of cartilaginous and proteoglycan-rich tissues throughout the body. The most commonly affected sites are the auricular pinna, nose, and joints with ocular tissue involvement occurring in up to 50% of patients. The most common ocular manifestations are scleritis, episcleritis, uveitis, and conjunctivitis. Less common ocular issues include keratitis, retinopathy, optic neuropathy, extraocular muscle palsy, and orbital inflammation. Due to the variable clinical presentation and rarity of the disease, the diagnosis of RPC is often delayed or it is misdiagnosed. It is important that ophthalmologists be aware of relapsing polychondritis because ocular symptoms may present as the initial manifestation of the disease.

## Introduction

The first case of relapsing polychondritis (RPC) was reported in 1923 by Dr. Jaksha-Walkenhorst. The current definition was proposed in 1960 by Pearson et al. [1] in which RPC was defined as a rare systemic autoimmune disease that causes episodes of inflammation throughout the body that can cause permanent destruction to the affected site over time. Inflammation most commonly involves cartilaginous or proteoglycan-rich tissues such as the external and internal ears, nose, joints, upper and lower respiratory tracts, eyes, heart, and blood vessels [2]. Patients with suspected RPC must present with inflammation of the cartilage in at least two locations or with a singular site of chondritis with at least two of the following symptoms: seronegative arthritis, ocular inflammation, hearing loss, and/or vestibular dysfunction [3]. While the prevalence of RPC is unknown, the incidence has been reported to be 3.5 cases per million people [2]. RPC is most commonly reported in Caucasian patients but can occur in all races, most commonly in the fourth decade of life [4]. No hereditary predisposition has been found, but a linkage to human leukocyte antigen-DR4 has been suggested [4]. The pathogenesis of RPC is elusive, possibly due to an autoimmune reaction against type II collagen, which plays a key role in humoral and cellular immunity. We present a case of acute angle closure in the setting of choroidal effusions and pan-scleritis due to relapsing polychondritis.

## Case presentation

A 52-year-old African American male with a past medical history significant for hypertension and chronic rhinitis with bilateral otitis externa for one week presented with eyelid irritation, injection, photophobia, and bilateral warmth and tenderness of the ears. The patient was seen by his primary care provider and prescribed antibacterial eye drops, which did not resolve his symptoms. Ophthalmology was consulted. The patient's vision on initial presentation was 20/20 in his right eye and 20/25 in his left eye with an intraocular pressure (IOP) of 18 by applanation in both eyes. He was noted to be a low hyperope with minimal astigmatism, previously correcting to 20/20 in both eyes (right eye +1.50 sphere, left eye +1.50 +1.00 x012). A slit lamp exam revealed superficial punctate keratitis, 2+ conjunctival injection, meibomian gland dysfunction, and trace nuclear sclerotic cataracts in both eyes but was otherwise unremarkable. The anterior chamber was deep and quiet without cell or flare in both eyes. Gonioscopy showed open angles in all quadrants bilaterally. Since the patient's symptoms had not resolved with antibacterial eye drops, his conjunctivitis was suspected to be either viral or allergic in nature. The patient was prescribed fluorometholone eye ointment twice a day with instructions to return in one week for follow-up.

**Figure 1:**
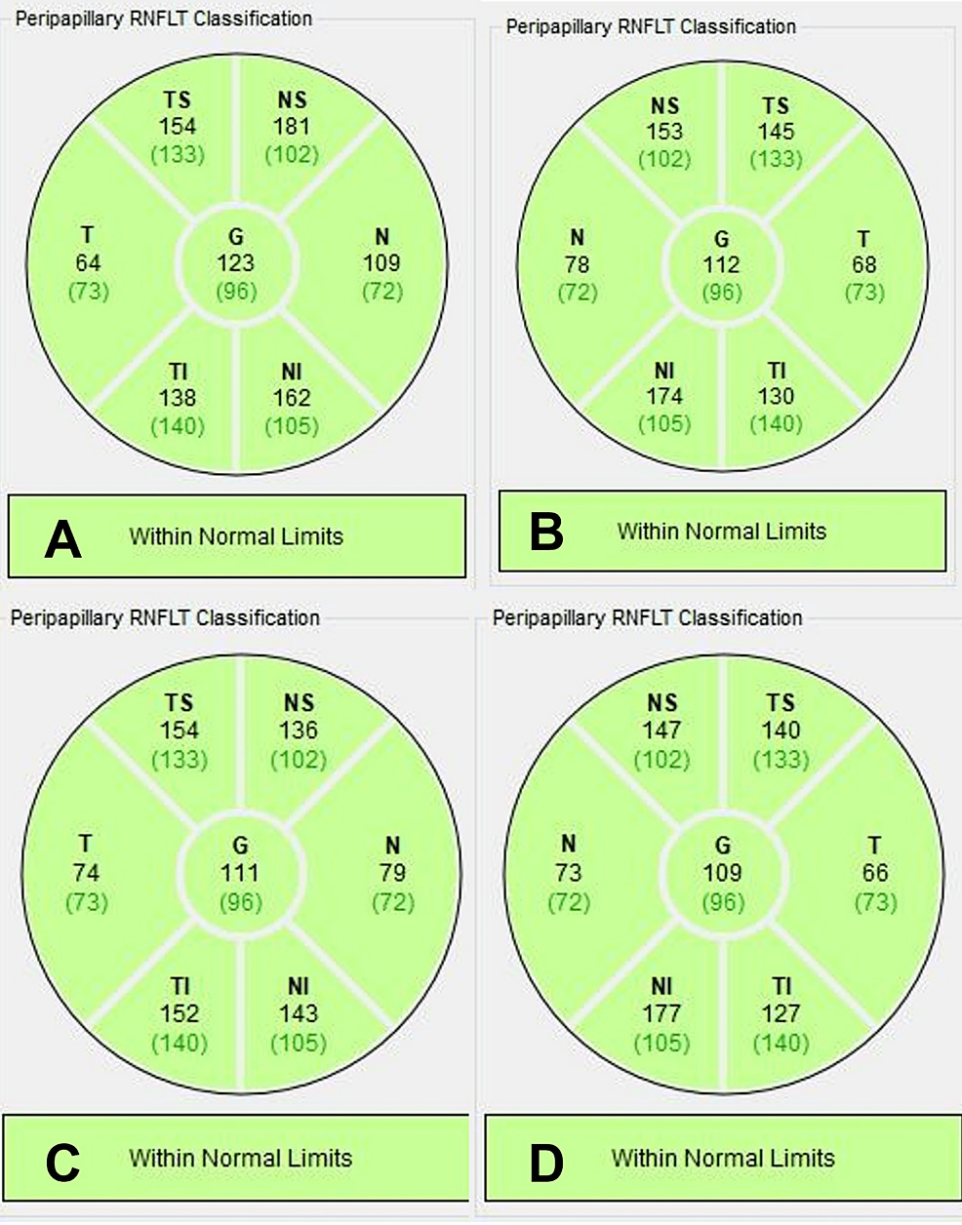
Optical Coherence Tomography (OCT) of the Retinal Nerve Fiber Layer (RNFL) A: Right eye at the time of angle closure diagnosis shows increased global, superior, and inferior thickening. B: Left eye at the time of angle closure diagnosis shows increased global, superior, and inferior thickening. C: Right eye one month after diagnosis shows improved global, superonasal, and inferonasal thickening. D: Left eye one month after diagnosis shows improved global and superior thickening.

Four days later, the patient returned to the clinic with worsening pain and photophobia bilaterally, which was more severe in the left eye. IOP by applanation was 16 in his right eye and 29 in his left eye. A slit lamp exam showed worsening conjunctival and scleral injection, likely causing the patient's pain. Repeated gonioscopy showed a completely closed angle (Shaffer grade 0) in his left eye. Optical coherence tomography (OCT) of the macula was unremarkable. OCT of the retinal nerve fiber layer (RNFL) showed global, superior, and inferior thickening in both eyes (Figure 1, A and B). The patient underwent bilateral lateral peripheral iridotomies (LPI) to relieve the angle closure in the left eye and for prophylaxis in the right eye.

**Figure 2:**
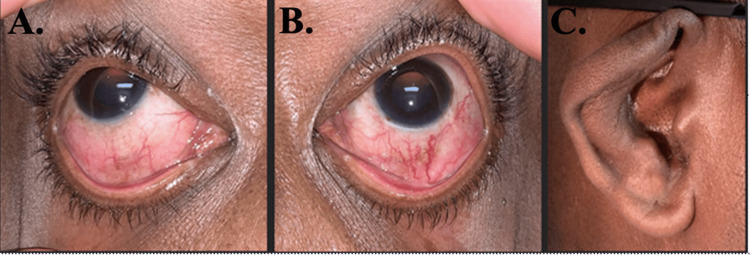
Diffuse anterior scleritis of the left and right eyes and post-inflammatory changes to the right ear A, B: Diffuse scleritis of the right and left eyes that did not blanch with the application of 2.5% phenylephrine. C: Post-inflammatory structural changes to the ear taken one week after the initiation of oral steroids.

**Figure 3:**
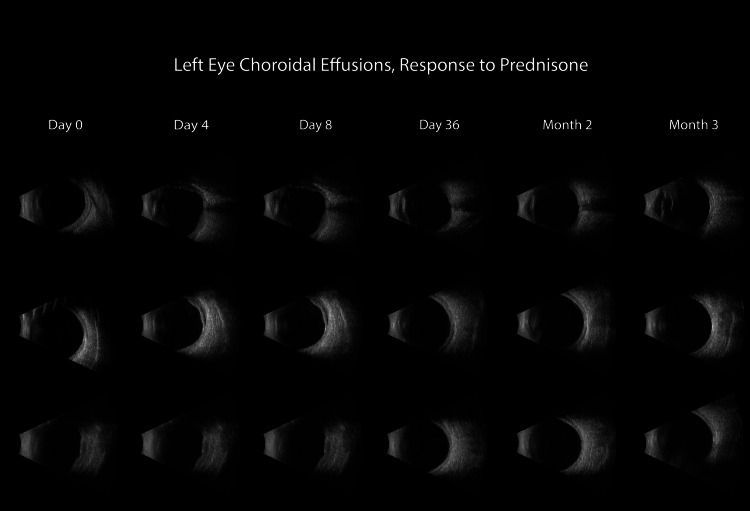
Serial B-scan ultrasound images illustrate the choroidal effusions found in the left eye Day 0 represents the day of prednisone initiation. While the choroidal effusions initially worsened over the first four days of treatment, near-complete resolution was achieved by day 8 and the patient had sustained resolution through month 3.

**Figure 4:**
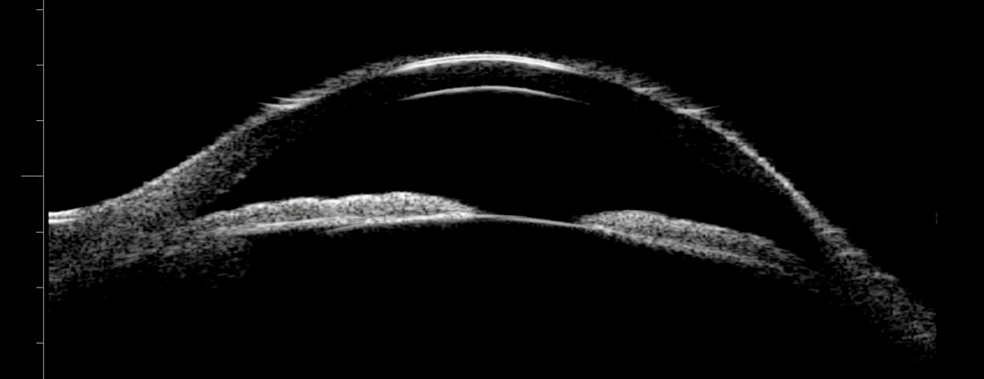
Ultrasound biomicroscopy (UBM) of the left eye showing an anterior displaced ciliary body and a narrow angle

Five days after undergoing bilateral LPI, the patient presented to the emergency department with worsening eye pain, injection, and photophobia. A slit lamp exam revealed diffuse anterior scleritis (Figure 2) and dilated fundus exam revealed low-lying anterior choroidal effusions. The patient was started on naproxen 500 mg twice per day, atropine three times per day in the left eye, and prednisolone acetate four times per day in both eyes. He was seen in the clinic the next day where B-scan ultrasound (10 MHz, Aviso, Quantel Medical, Minneapolis, Minnesota) (Figure 3) confirmed choroidal effusions as the etiology of the patient’s acute angle closure episodes. Ultrasound biomicroscopy (UBM, 50 MHz, Aviso) (Figure 4) of the left eye showed an anterior displaced ciliary body suggestive of angle narrowing.

The differential diagnosis at this time included relapsing polychondritis and autoimmune connective tissue disorders (including rheumatoid arthritis, systemic lupus erythematosus, granulomatosis with polyangiitis, polyarteritis nodosa, ankylosing spondylitis, sarcoidosis, Vogt-Koyanagi-Harada disease), infectious etiologies, and less likely to be idiopathic or due to LPI-associated trauma or medication use. Labs including CBC, comprehensive metabolic panel (CMP), erythrocyte sedimentation rate (ESR), C-reactive protein (CRP), uric acid, rapid plasma reagin (RPR), fluorescent treponemal antibody absorption test (FTA-ABS), antineutrophilic cytoplasmic antibody (ANCA), rheumatoid factor, anti-cyclic citrullinated peptide (CCP), antinuclear antibody (ANA), lysozyme, C3, C4, and urinalysis were ordered. The patient had an elevated white blood cell count of 22,000, ESR of 53, CRP of 64.6, and rheumatoid factor of 49. Urinalysis showed 2+ RBCs. All other labs were negative or within normal limits. The patient was presumptively diagnosed with relapsing polychondritis and referred to rheumatology.

Due to the severe presentation, the patient was started on oral prednisone 60 mg daily with proton pump inhibitor prophylaxis, and topical dorzolamide/timolol drops twice daily in both eyes for IOP control. Three weeks after the initiation of high-dose prednisone, the patient was seen by rheumatology and started on oral methotrexate. The starting dosage of methotrexate was 15 mg per week, which was gradually increased to 20 mg per week, yielding a favorable response with no significant side effects. The patient was also seen by otolaryngology to discuss the risks and benefits of a cartilage graft secondary to the destruction of helical cartilage bilaterally. While the option to have a cartilage graft was discussed, it was ultimately rejected due to the high possibility of recurrent inflammation and destruction of the helical cartilage. The patient reported mild hoarseness at the end of the day and carried a recent diagnosis of obstructive sleep apnea. A comprehensive upper airway examination was performed with a flexible fiberoptic scope, which revealed that the integrity of his laryngeal cartilage was minimally compromised; the patient elected to be observed.

The patient continued close follow-up with ophthalmology. Serial B-scan ultrasounds were performed until sustained resolution of the choroidal effusions was documented (Figure 3). Repeat OCT RNFL one month after angle closure showed improving RNFL thickness (Figure 1 C and D). He continued to show improvement with complete symptom resolution. His prednisone was slowly tapered by 10 mg decrease per month down to 5 mg daily while continuing his methotrexate. The patient’s presenting symptoms and response to treatment are consistent with an initial episode of pan-scleritis with choroidal effusions secondary to relapsing polychondritis. 

## Discussion

Patients with RPC can suffer from severe ocular manifestations including uveitis, conjunctivitis, and keratitis with diffuse anterior scleritis being the most common ocular manifestation [5]. Nodular, necrotizing, anterior, and posterior scleritis have also been observed [5]. Ocular symptoms occur in 20-61% of patients suffering from RPC [5]. When compared to scleritis caused by other systemic immune-mediated diseases, scleritis caused by RPC was more often bilateral, recurrent, necrotizing, and associated with visual disturbances [6].

Auricular chondritis is the most common symptom of RPC and develops in up to 89% of patients with RPC, frequently occurring as the presenting symptom in up to 26% of patients [4]. In 95% of cases, auricular chondritis is bilateral and presents with pain, warmth, erythema, and swelling of the cartilaginous portions of the ear [4]. During an episode of RPC, damage to the auricular cartilage may occur resulting in a flabby, droopy, or cauliflower ear [4].

The differential diagnosis of RPC includes systemic vasculitis, autoimmune conditions, trauma, and infection. Systemic vasculitis, such as granulomatosis with polyangiitis (GPA) and polyarteritis nodosa, may present similarly to RPC with necrotizing scleritis, keratitis, polyarteritis, vasculitis, and otitis media, but lack chondritis of the ear as seen in RPC [2]. Sarcoidosis may present with middle ear involvement and polyarthritis without chondritis [2]. Trauma and infection are typically unilateral and non-recurrent, although this can be difficult to assess in an initial encounter [2]. Our patient also lacked the classic systemic neurological (malaise, fever, nausea, neck/back stiffness, or tinnitus) and skin findings (alopecia, poliosis, or vitiligo) associated with VKH, nor has he developed late manifestations of VKH over the following year since presentation (Sugiura sign, chorioretinal scars [7].

Recent studies have suggested that vacuoles, E1 enzyme, X-linked, autoinflammatory, somatic (VEXAS) syndrome should be considered in patients with a clinical diagnosis of RPC [8]. VEXAS was first reported in 2020 by David Beck and colleagues from the National Institutes of Health and is a progressive autoinflammatory disease that presents with severe hematological and rheumatological conditions and is associated with a mutation in the UBA1 gene. Most notably, an increased risk of myelodysplastic syndrome has been identified in patients with VEXAS [8]. Due to the similarities in the clinical presentation of other autoimmune processes such as RPC, the diagnosis of VEXAS is challenging. Therefore, physicians should be aware of the potential association between VEXAS and RPC so that timely identification and treatment can occur, leading to better outcomes for affected individuals.

Due to the rarity of the disease, there have been no randomized clinical trials or evidence-based guidelines for the treatment of RPC [5]. Corticosteroids and immunologic agents that target ocular inflammation provide the most effective management in RPC with disease severity guiding medical management. In patients with mild cases, non-steroidal anti-inflammatory drugs (NSAIDs), dapsone, and colchicine can be used. In patients with severe disease progression, systemic corticosteroids can be used with immunosuppressants such as cyclophosphamide, methotrexate, azathioprine, and cyclosporine as second-line options for patients with contraindications to steroid use or requiring prolonged therapy for disease control [5].

Regarding the angle closure, our patient initially had open angles in both eyes, decreasing the likelihood of primary angle closure. He did not have peripheral anterior synechiae, neovascularization, a history of uveitis, a history of ocular surgery, nor an iris, ciliary body, or posterior mass, which can cause secondary angle closure. While he was phakic, he had a minimal nuclear sclerotic cataract development, ruling out a phacomorphic angle closure. The patient was not taking any anticholinergic or sympathomimetic medications associated with ciliary body swelling and suprachoroidal effusion. Given the development of choroidal effusion at the time of angle closure, the primary mechanism of angle closure appeared to be due to anterior ciliary body rotation and forward iris displacement. This was resolved with aqueous suppression (dorzolamide/timolol), LPI, pharmacological dilation (atropine), and high-dose oral steroids to treat the underlying pathology [9].

This case is particularly unique due to this patient's atypical presentation leading to a delay in diagnosis. Relapsing polychondritis is a rare disease and uncommonly presents with ocular symptoms in its initial presentation. Given its rarity, clinicians may not consider RPC as a possible diagnosis. This case serves to highlight the underlying pathophysiology, treatment strategies, and management of RPC. 

## Conclusions

Relapsing polychondritis is a rare systemic autoimmune disorder characterized by recurrent inflammation that leads to the destruction of cartilaginous and proteoglycan-rich tissues throughout the body. Due to the variable clinical presentations and rarity of the disease, RPC diagnosis is often delayed or misdiagnosed. This atypical presentation of RPC aims to contribute to the growing body and understanding of ocular manifestations in RPC. This case also serves as an example of how to assess and manage a patient with suspected RPC. As early diagnosis and prompt treatment improve patient prognosis, physicians should consider RPC in their differential when managing a patient who has ocular inflammation with systemic cartilaginous manifestations.
